# 3-D Terrain Node Coverage of Wireless Sensor Network Using Enhanced Black Hole Algorithm

**DOI:** 10.3390/s20082411

**Published:** 2020-04-23

**Authors:** Jeng-Shyang Pan, Qing-Wei Chai, Shu-Chuan Chu, Ning Wu

**Affiliations:** 1 College of Computer Science and Engineering, Shandong University of Science and Technology, Qingdao 266590, China; jspan@cc.kuas.edu.tw (J.-S.P.); mimanxiaowei@163.com (Q.-W.C.); 2 School of Electronic and Information Engineering, Beibu Gulf University, Qinzhou 535011, China; n.wu@bbgu.edu.cn

**Keywords:** node coverage, 3-D node deployed, WSN, intelligence computing, black hole

## Abstract

In this paper, a new intelligent computing algorithm named Enhanced Black Hole (EBH) is proposed to which the mutation operation and weight factor are applied. In EBH, several elites are taken as role models instead of only one in the original Black Hole (BH) algorithm. The performance of the EBH algorithm is verified by the CEC 2013 test suit, and shows better results than the original BH. In addition, the EBH and other celebrated algorithms can be used to solve node coverage problems of Wireless Sensor Network (WSN) in 3-D terrain with satisfactory performance.

## 1. Introduction

With the development of integrated circuits, micro-electromechanical systems (MEMS) and communication theory, the emergence of WSN has provided many conveniences for human life, and people can more conveniently monitor the surrounding environment or instruments easily. In WSN, optimizing the energy depletion ground on the limited size of sensor nodes while maintaining the data transmission rate is an enormous challenge. A self-adaptive clustering method is implemented to significantly save energy of sensor nodes [1]. Node coverage determines the detection property of WSN, and therefore achieving the maximum coverage rate with a given number of sensor nodes is of importance [2,3]. There are two methods to optimize sensor deployment: deterministic method and stochastic method. Deterministic method is used to deploy sensor nodes in a static environment for the predetermined positions of sensor nodes. The stochastic method randomly deploys sensor nodes with vehicles such as cars, planes, ships and et al. The deployment strategies for maximizing the coverage rate of WSN have not only been focused on the 2-D environment, but also on 3-D environment. Delaunay Triangulation and Voronoi Diagram have been used to find the sparse area of sensors and deploy new sensor nodes, which can significantly improve the coverage rate of WSN [4]. Bio-inspired algorithm has also been used to strengthen the capability of WSN [5], and PSO is applied to work out the energy-efficient coverage problem for the minimal cost [6]. Black Hole (BH) algorithm is an excellent meta-heuristic algorithm proposed in recent years [7]. It simulates the phenomenon that black holes engulf other planets in the universe. In the BH algorithm, the optimal solution is represented as the black hole and other candidate solutions are expressed as planets. The planet moves toward the black hole and finds the optimal solution on the way. If a planet is too close to a black hole, it will be engulfed by the black hole. Some simulations show that the BH algorithm has better performance in data clustering than some existing well-known algorithms.

Intelligent computing has been paid more and more attention by researchers because of its excellent performance in solving optimization problems, such as Artificial Bee Colony (ABC) [8,9,10], Particle Swarm Optimization (PSO) [11,12,13,14], Genetic Algorithm (GA) [15], Ant Colony Optimization (ACO) [16,17,18], Cat Swarm Optimization (CSO) [19,20,21], Difference Evolution (DE) [22,23,24], Multi-Verse Optimizer [25,26], Symbiotic Organism Search Algorithm [27,28], QUATRE [29,30,31] and et al. To improve the optimization efficiency, many methods have been put forward. For example, a compact method is implemented to achieve better performance based on the memory of a single individual [32,33,34,35,36,37]. The simulation of the benchmark function can also effectively improve the speed and sear-ability of the original algorithm [38]. In recent years, researchers are also inspired by the movement of animals to simulate the natural phenomena for optimization [39,40,41,42], and more and more improved algorithms have been proposed [43,44,45,46], including fuzzy processing [47,48,49].

Optimization methods are very important for increasing the efficiency of Wireless Sensor Network (WSN), which can efficiently monitor the object area at a low cost. In military applications, enemy invasion can be detected in real time by applying WSN. In agriculture applications, farmers can appropriately adjust the schedule or content of work after receiving the crop data obtained by the WSN. Moreover, we can prediction the localization, velocity and travel time of vehicle according to the information of sensor nodes gathered [50,51].

This article presents a novel intelligence computing algorithm named Enhanced Black Hole (EBH) to which the mutation operation and weight factor are applied. The BH algorithm and 3-D node coverage problem of WSN are recommended in Section 2. Section 3 proposes an Enhanced version of the EH algorithm, and Section 4 apply the novel algorithm in solving 3-D node coverage problem of WSN. Section 5 shows the experiments and the results. Finally, in Section 6 a conclusion is drawn.

## 2. Black Hole Algorithm and 3-D Node Coverage Problem of WSN

### 2.1. Black Hole Algorithm

Black Hole (BH) algorithm is an excellent optimization algorithm for solving data clustering problems, inspired by the phenomenon about black hole devouring other stars [7]. In BH algorithm, the individual with the best fitness value is regarded as a black hole, and other individuals move towards it. If the distance between an individual and the black hole is smaller than the radius of event horizon $R_{BH}$, then it will be swallowed by the black hole and an individual will be randomly generated to maintain the size of the population. As other population-based algorithms, at the initial stage, candidate solutions are generated randomly in a finite region, and then updates the population to find the optimal value according to Equation (1).

(1) $$\begin{array}{c} Pos_{i}^{t+1}=Pos_{i}^{t}+rand\cdot \left(Pos_{BH}^{t}-Pos_{i}^{t}\right) \end{array}$$

where the $Pos_{i}^{t}$ means the location of i-th candidate solution at the *t* iteration and $Pos_{BH}^{t}$ represents the black hole location at the *t* iteration. The rand is a random number between 0 and 1. From Equation (1) we can learn about the BH algorithm gives up the impact of $p_{best}$, velocity, and two constants. It has been found that the BH algorithm outperforms the PSO algorithm in data clustering [7], this can prove it is a meaningful algorithm in some ways.

In BH algorithm, the event horizon is used to describe the range of being devoured by the black hole and it can be calculated by Equation (2) such that

(2) $$\begin{array}{c} R_{BH}=\frac{fit_{BH}}{\sum_{n=1}^{N}fit_{n}} \end{array}$$

where $R_{BH}$ is the radius of event horizon and $fit_{BH}$ represents the best fitness value at the current iteration. The fitness value of n-t individual at the current iteration is represented by $fit_{n}$. The size of the population is represented by *N*. When the algorithm at an early stage, the population sparsely separates in the limited area. The black hole has the signally fitness value and the algorithm in the exploration phase. The longer the event horizon radius, the better to avoid the whole population being concentrated in one place and the algorithm precocious. On the contrary, when the algorithm in the exploitation phase, the algorithm needs to further look for the optimal value around the promising area.

### 2.2. 3-D Node Coverage Problem of WSN

In this study, EBH algorithm is introduced to optimize the strategy of sensor nodes deployed in a 3-D terrain. Various factors will be taken into account when deploying sensors to a 3-D terrain than a 2-D terrain. When a sensor node communicates with other nodes, the transmission of the wireless signal may be interrupted, and the coverage rate of WSN will be affected.

**Figure 1:**
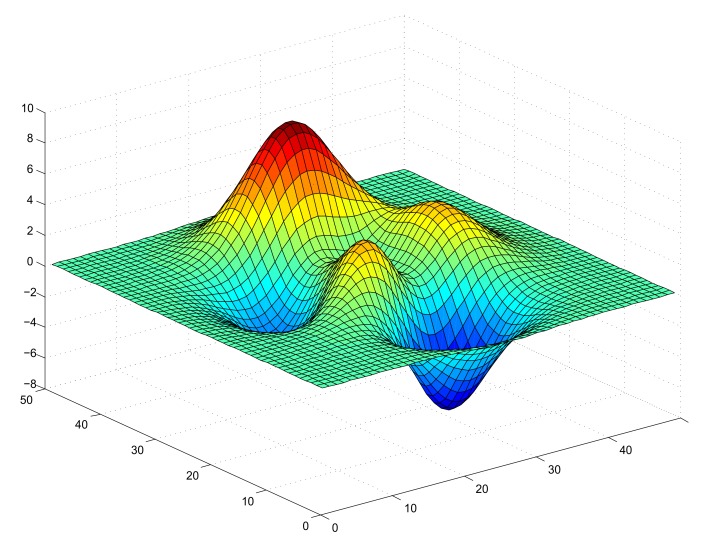
Terrain for Deploying Sensor Nodes (The units of x, y, z-axis is meter).

In the simulation, sensor nodes are scattered randomly over a 3-D terrain generated by the “peak” function of MATLAB as shown in Figure 1, and some sensor nodes are placed at the intersection of the grid. The 3-D locations of nodes in this 3-D terrain are defined by the initial allocation, and the depth locations are determined by the following rules:

- If the node is at the grid intersection, the value of the third dimension of the location is the height of the three-dimensional terrain which showed in Figure 1.
- Otherwise, the value of the third dimension of the location is the height of the closest grid intersection.

**Figure 2:**
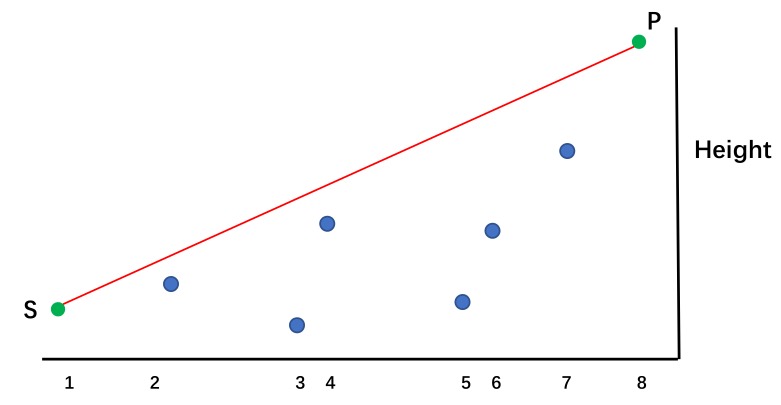
A Simple Paradigm about LOS.

In the cause of study, the node coverage of WSN in this 3-D terrain, it is necessary to detect whether there is an obstacle between two nodes. The Bresenham Line of Sight (LOS) algorithm is adopted for the optimization of this study thanks to its computational efficiency. To determine whether the communication between the node and the target point is blocked, some points between S to P are selected, as shown in Figure 2. If the height of any point in the terrain is higher than the ray height of the same point, the communication between S and P will be blocked [52].

In WSN, the sensor nodes collect the information about object and transmits to the sink node for further processing. The sensing model is formulated to describe the coverage rate and it is affected by the distance and other factors [53]. In this research, the binary sensing model is used to determine whether there is a connection between two nodes. Since this model only simulates the connectivity status of WSN, therefore it can only determine whether a sensor node is communicating with other nodes according to the communication radius. It can be presented by Equation (3), such that

(3) $$C\left(r,s\right)=\left\{\begin{array}{c} 1,distance(r,s)⩽R\;and\;there\;no\;obstacle\\ 0,distance(r,s)>R\;or\;there\;are\;obstacles \end{array}\right.$$

where the *r* and *s* represent receive node and send node, respectively. The communication radius is represented by *R*. If the value of C(r,s) is 1 means the node *r* can communicate with node *s* and vice versa.

In recent years, the Bresenham algorithm has been used in computer graphics to draw a line segment on a 2-D surface, in [53] the algorithm is modified and used it to determine LOS on a 3-D surface. Figure 2 shows a simplified paradigm about LOS on a 3-D terrain. It can be seen that if there is no pixel higher than the virtual line between node *S* and node *P*, these two nodes can communicate with each other, which means that there is a LOS between these two nodes.

## 3. Enhanced Black Hole Algorithm

BH algorithm is a competitive and novel algorithm, however its performance can be greatly improved. In BH algorithm, the motion of all individuals is only affected by the optimal solution position, and their trajectories are all straight lines. Therefore, there is not enough space for candidates to explore as they move towards the black hole. Although the BH algorithm has a rapid convergence rate and excellent performance in data clustering, it has a great chance to get stuck in the local optimal location. Therefore, a multiple black holes scheme has been considered, and the impact of the best candidate solution will be taken into account as well as the elite candidate solutions. In this study, we chose three best alternatives as models and applied varying degrees of gravity to other stars, such that

(4) $$\begin{array}{cc} Pos_{i}^{t+1}= & Pos_{i}^{t}+w\cdot [c_{1}\cdot \left(Pos_{BH1}^{t}-Pos_{i}^{t}\right)+c_{2}\cdot \left(Pos_{BH2}^{t}-Pos_{i}^{t}\right)\\ \; & +c_{3}\cdot \left(Pos_{BH3}^{t}-Pos_{i}^{t}\right)] \end{array}$$

where *w* represents the role of weight for each black hole, and $c_{1}$, $c_{2}$, and $c_{3}$ are three constants used to adjust the degree of influence of black holes. In the early stage, the individuals were sparsely distributed in the limited area, and the distances between the black holes and other stars are considerably longer than among the stars. Therefore, a heavier weight *w* can help to look for the optimal value in the promising area quicker. If a promising area is found by the algorithm, the optimal value will be targeted within a smaller area instead of moving over a wider area. Therefore, a smaller value of *w* will be suitable for this situation. Mutation operation is added to the original BH algorithm, which enhances the global search ability of the algorithm. In each iteration, the values of the three random dimensions of the first black hole fluctuate 20% up and down based on their values. If the new position of the first black hole is better than the original position, it will replace the original position, thereby affecting the population to find the optimal solution. In general, the best candidate solution will have the biggest impact on other stars and other elite candidate solutions have a smaller influence on other stars. The pseudo-code of the novel algorithm is shown in Algorithm 1.
**Algorithm 1**: The Enhanced Black Hole Algorithm
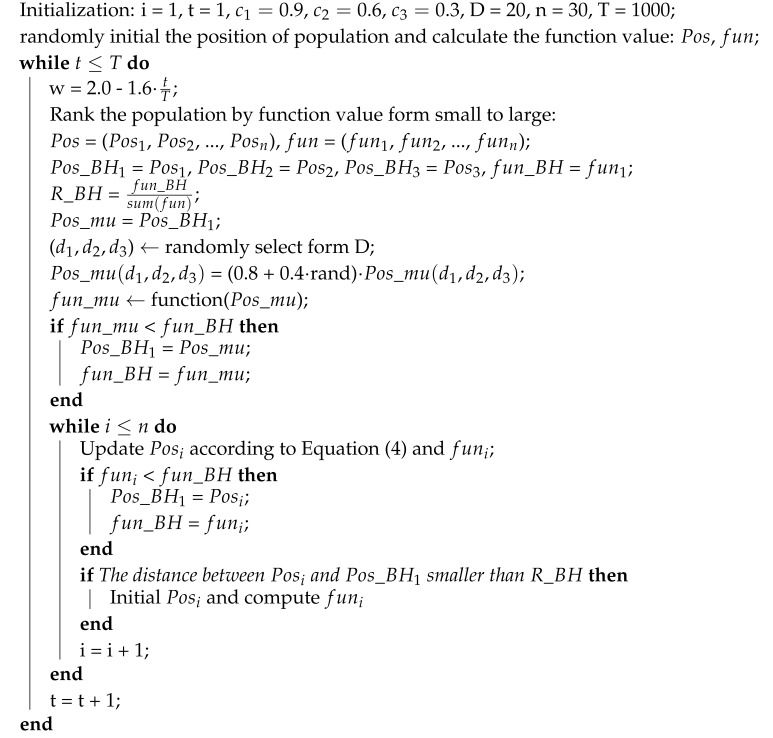


## 4. Enhanced Black Hole Algorithm Applied on Node Coverage of WSN

**Figure 3:**
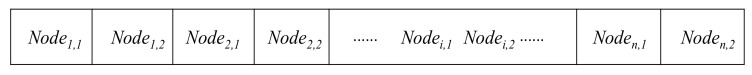
The Value Setting of Dimensions of Individual.

In the EBH algorithm, each individual represents a deployment strategy of the node coverage problem. Since an individual represents the deployment positions of all nodes, the dimension of each individual is the product of the dimensions of the deployment environment and the number of nodes. However, the 3-D positions of the deployed terrain have been known, if the position of the 2-D plane is provided, then the value of the depth location can be calculated. Figure 3 illustrates the setting of values for each individual in the EBH algorithm.

Where the $Node_{i,1}$ and $Node_{i,2}$ are the first dimension and second dimension value of the i-th sensor node. The *n* represents the amount of the deployed sensor nodes. In the initial stage, each individual is randomly generated, which means that each deployment strategy randomly arranges sensor nodes. Except for the optimal strategy, all deployment strategies learn from the optimal strategy according to the updated rules of the EBH algorithm. Coverage Rate (Cr) is defined to evaluate the quality of deployment strategies and is given as,

(5) $$Cr=\frac{1}{Q}\cdot \sum_{q=1}^{Q}\cdot \left(\sum_{p=1}^{P}C\left(s_{p},r_{q}\right)\right)$$

where the *Q* denotes the number of pixels of the 3-D terrain and *P* is the number of sensor nodes. Through $C(s_{p},r_{q})$, we can know whether the *q*-th pixel of the terrain is covered by the *p*-th sensor node and value of $C(s_{p},r_{q})$ can be calculated by Equation (3).

## 5. Experiment Results and Discuss

An improved BH algorithm is proposed in this paper. To verify the optimal performance of the algorithm, it is compared with the original algorithm using the CEC 2013 test suite. Moreover, the novel algorithm is compared with the canonical algorithm PSO and the state-of-the-art algorithm WOA. In the CEC 2013, there are 28 benchmark functions which are denoted as f1 to f28 respectively. There are some unimodal functions are the first five functions, f21 to f28 are composition functions and others are basic multimodal functions.

All experiments in this article are performed on the same platform which is a notebook computer with an i5-7300HQ CPU @ 2.50GHz. The parameters of the algorithm have an important impact on the results. To fairly verify the performance of the algorithms participating in the comparison, the parameters that they jointly own are set to the same value. The unique parameter values for these algorithms are set according to the recommendations of the original [7,11,40]. Table 1 shows the setting of the algorithm parameters.

Table 2 shows the average and standard deviation of the results of the 30 runs of the four algorithms on the CEC 2013 test suite. The results obtained by EBH are superior to other algorithms on functions 6, 10, 21, 22, 25 and especially significantly improves the results on functions 1, 3 and 26. In terms of stability, the proposed algorithm has better performance than other algorithm on functions 1, 5, 6, 10, 24, 25 and 26. The PSO algorithm is well suited to optimize problems contained in CEC2013 and it shows the best performance in most test functions. In these test functions, the WOA algorithm and the original BH algorithm are not sufficiently competitive with other algorithms.

Through the analysis of the results in Table 2, the EBH algorithm does not have the best processing effect on multimodal problems. As described in the “there is no free lunch” theorem [54], “any performance improvement for one type of problem will be offset by performance for another type of problem.” EBH wants to get better on unimodal problems, the performance of the multimodal problem will sacrifice some of the global search ability.

**Figure 4:**
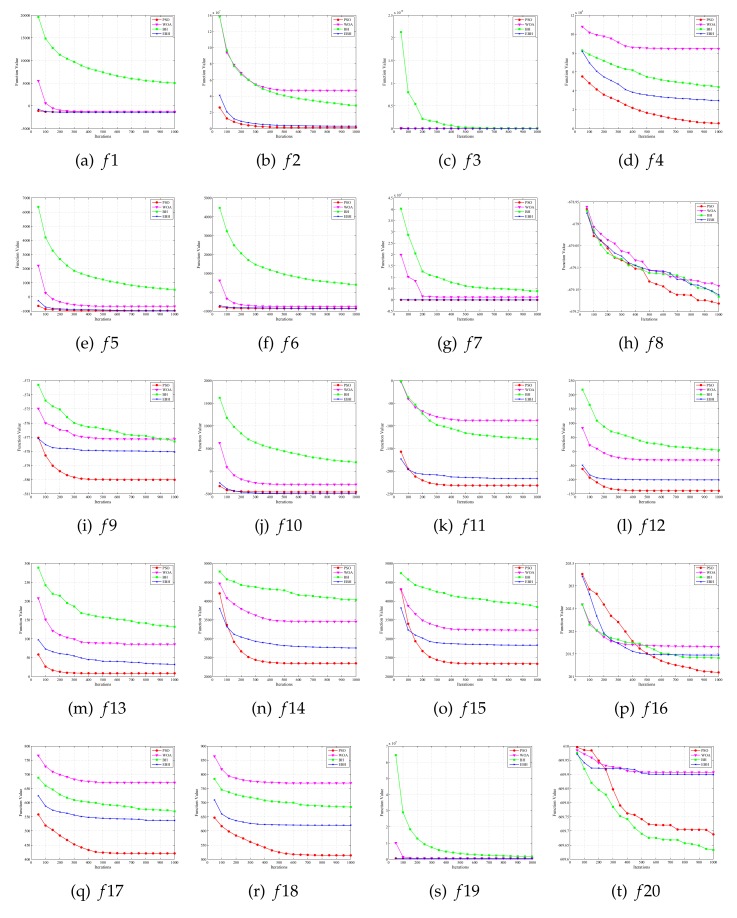
Results of Simulation Experiments (1).

**Figure 5:**
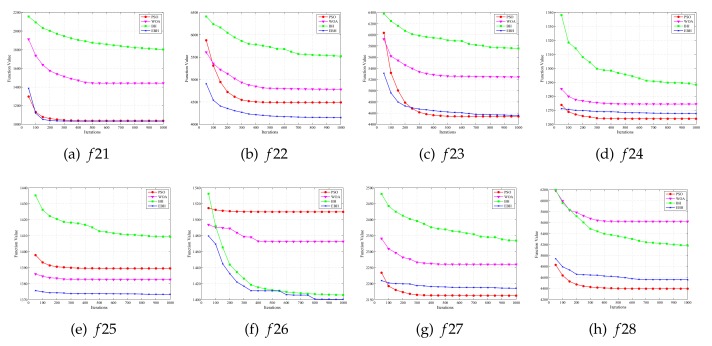
Results of Simulation Experiments (2).

Figure 4 and Figure 5 show the process of optimization of four algorithms on 28 test functions. The abscissa is the number of iterations of the algorithm running, and the ordinate is the average value obtained from the optimization test function 30 times, a function value is recorded for every 100 iterations to make a graph to prove the search ability of the algorithm. The PSO, WOA, BH, and EBH algorithms are represented by “red”, “magenta”, “green” and “blue” respectively. In these figures, the EBH algorithm has faster convergence speed and stronger optimization ability than the original algorithm on unimodal problems, composition problems and most multimodal problems. Although the EBH algorithm has fast convergence speed, it is easily trapped at local optimal value on function 8 and 20. Therefore the original algorithm has better performance on these two functions.

In this article, the novel algorithm is used to solve the node coverage problem of WSN. This algorithm optimizes the positions of sensor nodes to achieve maximize coverage area with same account of nodes. The sensor nodes in the WSN are arranged in the terrain shown in Figure 1 and are tested in five groups of different number of nodes, which are 30, 40, 50, 60, and 70 nodes, respectively. In this simulation experiment, the area of plane of the terrain is 50 × 50 m and the communication radius is set to 5 m for each group.

Table 3 shows the results of 5 sets of simulations, which are the average of 30 runs. The data of different groups shows the influence of the number of nodes on the optimization results. When the communication radius is constant, the more nodes, the larger the area covered. Therefore, the difference between the optimization results of different algorithms decreases as the number of nodes increases. In Table 3, the new algorithm performs best on 30, 40, 50, and 70 sensor nodes. Although the difference is relatively small, in this optimization problem with few dimensions, we can still see the excellent performance of the new algorithm.

## 6. Conclusions

In this paper, we enhanced the BH algorithm by considering more influencing factors and putting weight to the optimization of BH algorithm. Compared to the original BH algorithm, the EBH achieves a greater probability of falling into a promising region and avoiding local optimal values with a faster convergence speed. In the experiment with the CEC 2013 benchmark functions, the EBH algorithm shows better performance than the original BH in all test functions. The EBH algorithm has also been proved to be effective in solving the 3-D node coverage problems in WSN. The proposed EBH algorithm can also be applied to solve the cluster head problem of WSN in future.

## Figures and Tables

**Table 1:** Parameter Setting of Algorithms.

Algorithms	Common Parameters	Unique Parameters
Particle Swarm Optimization	Population Size = 30 Iterations = 1000 Dimensions = 20 Limited Areas ∈ [−100, 100]	c = 2.0, w ∈ [0.4, 0.9], Velocity Range ∈ [10, 10]
Whale Optimization Algorithms		a ∈ [0, 2], b = 1
Black Hole		NULL
Enhanced Black Hole		w ∈ [0.4, 2.0], $c_{1}$ = 0.9, $c_{2}$ = 0.6, $c_{3}$ = 0.3

**Table 2:** Simulation Results of CEC 2013 Benchmark Function (The optimal value is marked by bold).

Functions	PSO		WOA		BH		EBH	
**Variable**	**Mean**	**Std**	**Mean**	**Std**	**Mean**	**Std**	**Mean**	**Std**
f1	$-1.36\times 10^{3}$	$1.58\times 10^{3}$	$-1.29\times 10^{3}$	$8.43\times 10^{1}$	$5.04\times 10^{3}$	$1.02\times 10^{3}$	$-1.40\times 10^{3}$	$9.51\times 10^{-7}$
f2	$1.22\times 10^{6}$	$7.61\times 10^{5}$	$4.69\times 10^{7}$	$2.25\times 10^{7}$	$2.84\times 10^{7}$	$4.47\times 10^{6}$	$2.92\times 10^{6}$	$1.62\times 10^{6}$
f3	$2.92\times 10^{9}$	$4.08\times 10^{9}$	$2.14\times 10^{10}$	$3.47\times 10^{10}$	$3.54\times 10^{15}$	$4.78\times 10^{15}$	$5.91\times 10^{8}$	$8.24\times 10^{8}$
f4	$5.55\times 10^{3}$	$3.47\times 10^{3}$	$8.46\times 10^{4}$	$2.99\times 10^{4}$	$4.39\times 10^{4}$	$1.20\times 10^{4}$	$2.96\times 10^{4}$	$8.27\times 10^{3}$
f5	$-9.68\times 10^{2}$	$5.26\times 10^{1}$	$-6.67\times 10^{2}$	$1.64\times 10^{2}$	$5.08\times 10^{2}$	$2.54\times 10^{2}$	$-9.69\times 10^{2}$	$2.73\times 10^{1}$
f6	$-8.54\times 10^{2}$	$3.73\times 10^{1}$	$-7.62\times 10^{2}$	$6.73\times 10^{1}$	$3.99\times 10^{2}$	$1.48\times 10^{2}$	$-8.61\times 10^{2}$	$3.71\times 10^{1}$
f7	$-7.18\times 10^{2}$	$4.16\times 10^{1}$	$1.18\times 10^{4}$	$6.04\times 10^{4}$	$3.84\times 10^{4}$	$3.25\times 10^{4}$	$-4.84\times 10^{2}$	$4.05\times 10^{2}$
f8	$-6.79\times 10^{2}$	$8.05\times 10^{-2}$	$-6.79\times 10^{2}$	$8.41\times 10^{-2}$	$-6.79\times 10^{2}$	$6.87\times 10^{-2}$	$-6.79\times 10^{2}$	$8.27\times 10^{-2}$
f9	$-5.80\times 10^{2}$	$2.87\times 10^{0}$	$-5.77\times 10^{2}$	$1.93\times 10^{0}$	$-5.77\times 10^{2}$	$2.29\times 10^{0}$	$-5.78\times 10^{2}$	$2.51\times 10^{0}$
f10	$-4.60\times 10^{2}$	$6.67\times 10^{1}$	$-2.95\times 10^{2}$	$7.51\times 10^{1}$	$1.97\times 10^{2}$	$7.37\times 10^{1}$	$-4.98\times 10^{2}$	$1.49\times 10^{0}$
f11	$-2.32\times 10^{2}$	$4.95\times 10^{1}$	$-8.97\times 10^{1}$	$7.55\times 10^{1}$	$-1.29\times 10^{2}$	$4.63\times 10^{1}$	$-2.16\times 10^{2}$	$4.90\times 10^{1}$
f12	$-1.39\times 10^{2}$	$4.80\times 10^{1}$	$-3.05\times 10^{1}$	$5.40\times 10^{1}$	$4.53\times 10^{0}$	$6.03\times 10^{1}$	$-1.01\times 10^{2}$	$5.57\times 10^{1}$
f13	$8.59\times 10^{0}$	$2.98\times 10^{1}$	$8.56\times 10^{1}$	$7.01\times 10^{1}$	$1.32\times 10^{2}$	$5.77\times 10^{1}$	$3.19\times 10^{1}$	$5.36\times 10^{1}$
f14	$2.35\times 10^{3}$	$4.36\times 10^{2}$	$3.45\times 10^{3}$	$4.64\times 10^{2}$	$4.03\times 10^{3}$	$5.65\times 10^{2}$	$2.76\times 10^{3}$	$5.77\times 10^{2}$
f15	$2.34\times 10^{3}$	$5.51\times 10^{2}$	$3.23\times 10^{3}$	$5.74\times 10^{2}$	$3.84\times 10^{3}$	$5.81\times 10^{2}$	$2.83\times 10^{3}$	$5.79\times 10^{2}$
f16	$2.01\times 10^{2}$	$3.85\times 10^{-1}$	$2.02\times 10^{2}$	$3.70\times 10^{-1}$	$2.01\times 10^{2}$	$4.66\times 10^{-1}$	$2.01\times 10^{2}$	$7.65\times 10^{-1}$
f17	$4.21\times 10^{2}$	$2.64\times 10^{1}$	$6.71\times 10^{2}$	$8.93\times 10^{1}$	$5.69\times 10^{2}$	$5.05\times 10^{1}$	$5.37\times 10^{2}$	$5.87\times 10^{1}$
f18	$5.14\times 10^{2}$	$2.44\times 10^{1}$	$7.69\times 10^{2}$	$5.75\times 10^{1}$	$6.85\times 10^{2}$	$5.94\times 10^{1}$	$6.20\times 10^{2}$	$6.39\times 10^{1}$
f19	$5.07\times 10^{2}$	$2.39\times 10^{0}$	$5.49\times 10^{2}$	$2.58\times 10^{1}$	$1.70\times 10^{3}$	$3.96\times 10^{2}$	$5.13\times 10^{2}$	$5.90\times 10^{0}$
f20	$6.10\times 10^{2}$	$2.38\times 10^{-1}$	$6.10\times 10^{2}$	$1.87\times 10^{-1}$	$6.10\times 10^{2}$	$1.63\times 10^{-1}$	$6.10\times 10^{2}$	$2.02\times 10^{-1}$
f21	$1.04\times 10^{3}$	$8.26\times 10^{1}$	$1.44\times 10^{3}$	$2.50\times 10^{2}$	$1.80\times 10^{3}$	$5.40\times 10^{1}$	$1.03\times 10^{3}$	$9.43\times 10^{1}$
f22	$4.49\times 10^{3}$	$5.69\times 10^{2}$	$4.78\times 10^{3}$	$5.28\times 10^{2}$	$5.52\times 10^{3}$	$4.74\times 10^{2}$	$4.15\times 10^{3}$	$7.89\times 10^{2}$
f23	$4.54\times 10^{3}$	$5.98\times 10^{2}$	$5.25\times 10^{3}$	$6.81\times 10^{2}$	$5.76\times 10^{3}$	$5.01\times 10^{2}$	$4.56\times 10^{3}$	$8.32\times 10^{2}$
f24	$1.26\times 10^{3}$	$1.85\times 10^{1}$	$1.27\times 10^{3}$	$6.50\times 10^{0}$	$1.29\times 10^{3}$	$8.80\times 10^{0}$	$1.27\times 10^{3}$	$8.77\times 10^{0}$
f25	$1.39\times 10^{3}$	$1.24\times 10^{1}$	$1.38\times 10^{3}$	$7.81\times 10^{0}$	$1.41\times 10^{3}$	$9.02\times 10^{0}$	$1.37\times 10^{3}$	$6.63\times 10^{0}$
f26	$1.51\times 10^{3}$	$6.65\times 10^{1}$	$1.47\times 10^{3}$	$8.01\times 10^{1}$	$1.41\times 10^{3}$	$2.08\times 10^{0}$	$1.40\times 10^{3}$	$4.42\times 10^{-1}$
f27	$2.16\times 10^{3}$	$7.73\times 10^{1}$	$2.26\times 10^{3}$	$5.24\times 10^{1}$	$2.33\times 10^{3}$	$6.09\times 10^{1}$	$2.19\times 10^{3}$	$6.32\times 10^{1}$
f28	$4.39\times 10^{3}$	$6.63\times 10^{2}$	$5.62\times 10^{3}$	$7.61\times 10^{2}$	$5.18\times 10^{3}$	$5.63\times 10^{2}$	$4.56\times 10^{3}$	$5.90\times 10^{2}$

**Table 3:** Simulation Results of Node Coverage (The optimal value is marked by bold).

Functions	PSO	WOA	BH	EBH
30	45.55%	47.88%	47.82%	**48.01%**
40	55.53%	57.43%	57.75%	**57.85%**
50	62.88%	62.51%	64.99%	**65.06%**
60	69.44%	**71.50%**	71.32%	71.26%
70	74.71%	76.43%	76.43%	**76.54%**

## Abbreviations

The following abbreviations are used in this manuscript:

LD	Linear dichroism
MEMS	Micro-electro mechanical systems
PSO	Particle swarm optimization
ACO	Ant colony optimization
CSO	Cat swarm optimization
ABC	Artificial bee colony
GA	Genetic algorithm
DE	Difference evolution
QUATRE	Quasi-affine transformation evolution
WSN	Wireless sensor network
EBH	Enhanced black hole
BH	Black hole
LOS	Line of sight

## References

[1] Wang J., Gao Y., Wang K., Sangaiah A.K., Lim S.J. (2019). An Affinity Propagation-Based Self-Adaptive Clustering Method for Wireless Sensor Networks. Sensors.

[2] Tsang Y.P., Choy K.L., Wu C.H., Ho G.T.S. (2019). Multi-objective mapping method for 3D environmental sensor network deployment. IEEE Commun. Lett..

[3] Wang J., Ju C., Kim H.J., Sherratt R.S., Lee S. (2019). A mobile assisted coverage hole patching scheme based on particle swarm optimization for WSNs. Clust. Comput..

[4] Wu C.H., Lee K.C., Chung Y.C. (2007). A Delaunay triangulation based method for wireless sensor network deployment. Comput. Commun..

[5] Kulkarni R.V., Venayagamoorthy G.K. (2010). Bio-inspired algorithms for autonomous deployment and localization of sensor nodes. IEEE Trans. Syst. Man Cybern. Part C Appl. Rev..

[6] Wang J., Ju C., Gao Y., Sangaiah A.K., Kim G.J. (2018). A PSO based energy efficient coverage control algorithm for wireless sensor networks. Comput. Mater. Contin..

[7] Hatamlou A. (2013). Black hole: A new heuristic optimization approach for data clustering. Inf. Sci..

[8] Karaboga D. (2005). An Idea Based on Honey Bee Swarm for Numerical Optimization.

[9] TSai P.W., Pan J.S., Liao B.Y., Chu S.C. (2009). Enhanced artificial bee colony optimization. Int. J. Innov. Comput. Inf. Control.

[10] Wang H., Wu Z., Rahnamayan S., Sun H., Liu Y., Pan J.S. (2014). Multi-strategy ensemble artificial bee colony algorithm. Inf. Sci..

[11] Eberhart R., Kennedy J. (1995). Particle swarm optimization. Proc. IEEE Int. Conf. Neural Netw. Citeseer.

[12] Liang J.J., Qin A.K., Suganthan P.N., Baskar S. (2006). Comprehensive learning particle swarm optimizer for global optimization of multimodal functions. IEEE Trans. Evol. Comput..

[13] Wang H., Wang W., Wu Z. (2013). Particle swarm optimization with adaptive mutation for multimodal optimization. Appl. Math. Comput..

[14] Melin P., Olivas F., Castillo O., Valdez F., Soria J., Valdez M. (2013). Optimal design of fuzzy classification systems using PSO with dynamic parameter adaptation through fuzzy logic. Expert Syst. Appl..

[15] Whitley D. (1994). A genetic algorithm tutorial. Stat. Comput..

[16] Dorigo M., Birattari M., Stutzle T. (2006). Ant colony optimization. IEEE Comput. Intell. Mag..

[17] Chu S.C., Roddick J.F., Pan J.S. (2004). Ant colony system with communication strategies. Inf. Sci..

[18] Chu S.C., Roddick J.F., Su C.J., Pan J.S. (2004). Constrained ant colony optimization for data clustering. Proceedings of the Pacific Rim International Conference on Artificial Intelligence.

[19] Chu S.C., Tsai P.W., Pan J.S. (2006). Cat swarm optimization. Proceedings of the Pacific Rim International Conference on Artificial Intelligence.

[20] Tsai P.W., Pan J.S., Chen S.M., Liao B.Y., Hao S.P. Parallel cat swarm optimization. Proceedings of the 2008 International Conference on Machine Learning and Cybernetics.

[21] Tsai P.W., Pan J.S., Chen S.M., Liao B.Y. (2012). Enhanced parallel cat swarm optimization based on the Taguchi method. Expert Syst. Appl..

[22] Storn R., Price K. (1997). Differential evolution–a simple and efficient heuristic for global optimization over continuous spaces. J. Glob. Optim..

[23] Pan J.S., Liu N., Chu S.C. (2020). A Hybrid Differential Evolution Algorithm and Its Application in Unmanned Combat Aerial Vehicle Path Planning. IEEE Access.

[24] Meng Z., Pan J.S., Tseng K.K. (2019). PaDE: An enhanced Differential Evolution algorithm with novel control parameter adaptation schemes for numerical optimization. Knowl. Based Syst..

[25] Mirjalili S., Mirjalili S.M., Hatamlou A. (2016). Multi-verse optimizer: A nature-inspired algorithm for global optimization. Neural Comput. Appl..

[26] Wang X., Pan J.S., Chu S.C. (2020). A Parallel Multi-Verse Optimizer for Application in Multilevel Image Segmentation. IEEE Access.

[27] Ezugwu A.E., Prayogo D. (2019). Symbiotic Organisms Search Algorithm: Theory, recent advances and applications. Expert Syst. Appl..

[28] Chu S.C., Du Z.G., Pan J.S. (2020). Symbiotic Organism Search Algorithm with Multi-Group Quantum-Behavior Communication Scheme Applied in Wireless Sensor Networks. Appl. Sci..

[29] Meng Z., Pan J.S. QUasi-affine TRansformation Evolutionary (QUATRE) algorithm: A parameter-reduced differential evolution algorithm for optimization problems. Proceedings of the 2016 IEEE Congress on Evolutionary Computation (CEC).

[30] Meng Z., Pan J.S. A competitive QUasi-Affine TRansformation Evolutionary (C-QUATRE) algorithm for global optimization. Proceedings of the 2016 IEEE International Conference on Systems, Man, and Cybernetics (SMC).

[31] Liu N., Pan J.S., Xue J.Y. (2020). An Orthogonal QUasi-Affine TRansformation Evolution (O-QUATRE) Algorithm for Global Optimization. Advances in Intelligent Information Hiding and Multimedia Signal Processing.

[32] Harik G.R., Lobo F.G., Goldberg D.E. (1999). The compact genetic algorithm. IEEE Trans. Evol. Comput..

[33] Dao T.K., Pan J.S., Nguyen T.T., Chu S.C., Shieh C.S. (2014). Compact bat algorithm. Intelligent Data Analysis and Its Applications, Volume II.

[34] Tian A.Q., Chu S.C., Pan J.S., Cui H., Zheng W.M. (2020). A Compact Pigeon-Inspired Optimization for Maximum Short-Term Generation Mode in Cascade Hydroelectric Power Station. Sustainability.

[35] Xue X., Chen J. A Compact co-Firefly Algorithm for Matching Ontologies. Proceedings of the 2019 IEEE Symposium Series on Computational Intelligence (SSCI).

[36] Xue X., Pan J.S. (2018). A Compact Co-Evolutionary Algorithm for sensor ontology meta-matching. Knowl. Inf. Syst..

[37] Chu S.C., Xue X., Pan J.S., Wu X. (2020). Optimizing ontology alignment in vector space. J. Internet Technol..

[38] Sun C., Jin Y., Cheng R., Ding J., Zeng J. (2017). Surrogate-assisted cooperative swarm optimization of high-dimensional expensive problems. IEEE Trans. Evol. Comput..

[39] Mirjalili S., Mirjalili S.M., Lewis A. (2014). Grey wolf optimizer. Adv. Eng. Softw..

[40] Mirjalili S., Lewis A. (2016). The whale optimization algorithm. Adv. Eng. Softw..

[41] Duan H., Qiao P. (2014). Pigeon-inspired optimization: A new swarm intelligence optimizer for air robot path planning. Int. J. Intell. Comput. Cybern..

[42] Yang X.S. (2010). A new metaheuristic bat-inspired algorithm. Nature Inspired Cooperative Strategies for Optimization (NICSO 2010).

[43] Hu P., Pan J.S., Chu S.C., Chai Q.W., Liu T., Li Z.C. (2019). New Hybrid Algorithms for Prediction of Daily Load of Power Network. Appl. Sci..

[44] Chai Q.W., Chu S.C., Pan J.S., Hu P., Zheng W.M. (2020). A parallel WOA with two communication strategies applied in DV-Hop localization method. EURASIP J. Wirel. Commun. Netw..

[45] Pan J.S., Hu P., Chu S.C. (2019). Novel Parallel Heterogeneous Meta-Heuristic and Its Communication Strategies for the Prediction of Wind Power. Processes.

[46] Nguyen T.T., Pan J.S., Dao T.K. (2019). An improved flower pollination algorithm for optimizing layouts of nodes in wireless sensor network. IEEE Access.

[47] Gustafson D.E., Kessel W.C. Fuzzy clustering with a fuzzy covariance matrix. Proceedings of the 1978 IEEE Conference on Decision and Control Including the 17th Symposium on Adaptive Processes.

[48] Chen S.M., Manalu G.M.T., Pan J.S., Liu H.C. (2013). Fuzzy forecasting based on two-factors second-order fuzzy-trend logical relationship groups and particle swarm optimization techniques. IEEE Trans. Cybern..

[49] Chen S.M., Chang Y.C., Pan J.S. (2012). Fuzzy rules interpolation for sparse fuzzy rule-based systems based on interval type-2 Gaussian fuzzy sets and genetic algorithms. IEEE Trans. Fuzzy Syst..

[50] Chen C.H., Lee C.A., Lo C.C. (2016). Vehicle localization and velocity estimation based on mobile phone sensing. IEEE Access.

[51] Chen C.H., Hwang F.J., Kung H.Y. (2019). Travel time prediction system based on data clustering for waste collection vehicles. IEICE Trans. Inf. Syst..

[52] Topcuoglu H.R., Ermis M., Sifyan M. (2010). Positioning and utilizing sensors on a 3-D terrain part I—Theory and modeling. IEEE Trans. Syst. Man Cybern. Part C Appl. Rev..

[53] Temel S., Unaldi N., Kaynak O. (2013). On deployment of wireless sensors on 3-D terrains to maximize sensing coverage by utilizing cat swarm optimization with wavelet transform. IEEE Trans. Syst. Man Cybern. Syst..

[54] Wolpert D.H., Macready W.G. (1997). No free lunch theorems for optimization. IEEE Trans. Evol. Comput..

